# Carcinoid Heart Disease Management: A Multi-Disciplinary Collaboration

**DOI:** 10.1093/oncolo/oyad126

**Published:** 2023-05-20

**Authors:** Satya Das, Shannon S Stockton, Saamir A Hassan

**Affiliations:** Late Development Oncology, GI, AstraZeneca, Gaithersburg, MD, USA; Division of Hematology and Oncology, Department of Medicine, Vanderbilt University Medical Center, Nashville, TN, USA; Ascension Texas Cardiovascular, Cedar Park, TX, USA

**Keywords:** carcinoid heart disease, 5-hydroxyindoleacetic acid, N-terminal pro B-type natriuretic peptide, carcinoid syndrome, neuroendocrine tumors

## Abstract

Carcinoid heart disease (CaHD) is an important complication among patients with metastatic neuroendocrine tumors and carcinoid syndrome (CS). CS patients (25%-65%) eventually develop CaHD; these patients face a significantly increased risk of morbidity and mortality. Guidance papers (eg, clinical practice guidelines, consensus guidelines, and expert statements) have been established by major organizations across the disciplines of cardiology and oncology; however, these recommendations are not routinely implemented. The aim of this article is to encourage the integration of current recommendations from national societies into clinical practice. Early screening upon recognition of CS and prior to the development of CaHD symptoms is paramount, as no existing therapies are approved to reverse the fibrotic damage to the heart once it occurs. Valvular replacement is the only definitive treatment for CaHD once it has developed. When patients are noted to have urinary 5-hydroxyindoleacetic acid (5-HIAA) levels ≥300 µmol/24 h and/or serum N-terminal pro B-type natriuretic peptide (NT-proBNP) levels >260 pg/mL, echocardiography is recommended. Systemic approaches to control tumor growth and hormonal secretion include somatostatin analogs (SSAs), followed by options including peptide receptor radiotherapy (PRRT), everolimus and liver embolization. Telotristat is the primary choice for control of diarrhea refractory to SSA. Diuretics are the mainstay of heart failure symptom management for patients who develop CaHD. Considerations for future research are discussed, including the ongoing TELEHEART (TELotristat Ethyl in a HEART biomarker study) trial involving telotristat and not yet activated CHARRT (Carcinoid Heart disease And peptide Receptor Radiotargetted Therapy) study involving PRRT with lutetium 177 (^177^Lu) dotatate.

## Teaser

Carcinoid heart disease (CaHD) is a complication for patients with metastatic neuroendocrine tumors and carcinoid syndrome. This article reviews major CaHD guidance papers and provides expert clinical perspectives on how to implement these recommendations into clinical practice, with the goal of increasing screening for CaHD, optimizing multidisciplinary collaboration, and discussing exploratory therapies to prevent the development of CaHD.

**Implications for Practice**

CaHD is a devastating and frequent complication for NET patients with CS; approximately 25%-65% of CS patients develop CaHD during their disease course. Patients with untreated CaHD develop inevitable heart failure and possess 3-year survival rates as low as 31%. Timely screening and early diagnosis of CaHD are essential, as there are no therapies that can reverse fibrotic damage to the heart. It is imperative that clinicians caring for NET patients with CS routinely monitor 5-HIAA levels and/or NT-proBNP levels to identify patients at risk for developing CaHD; if CaHD is diagnosed, early referral to multidisciplinary NET centers is crucial.

## Introduction

**Figure 1.**
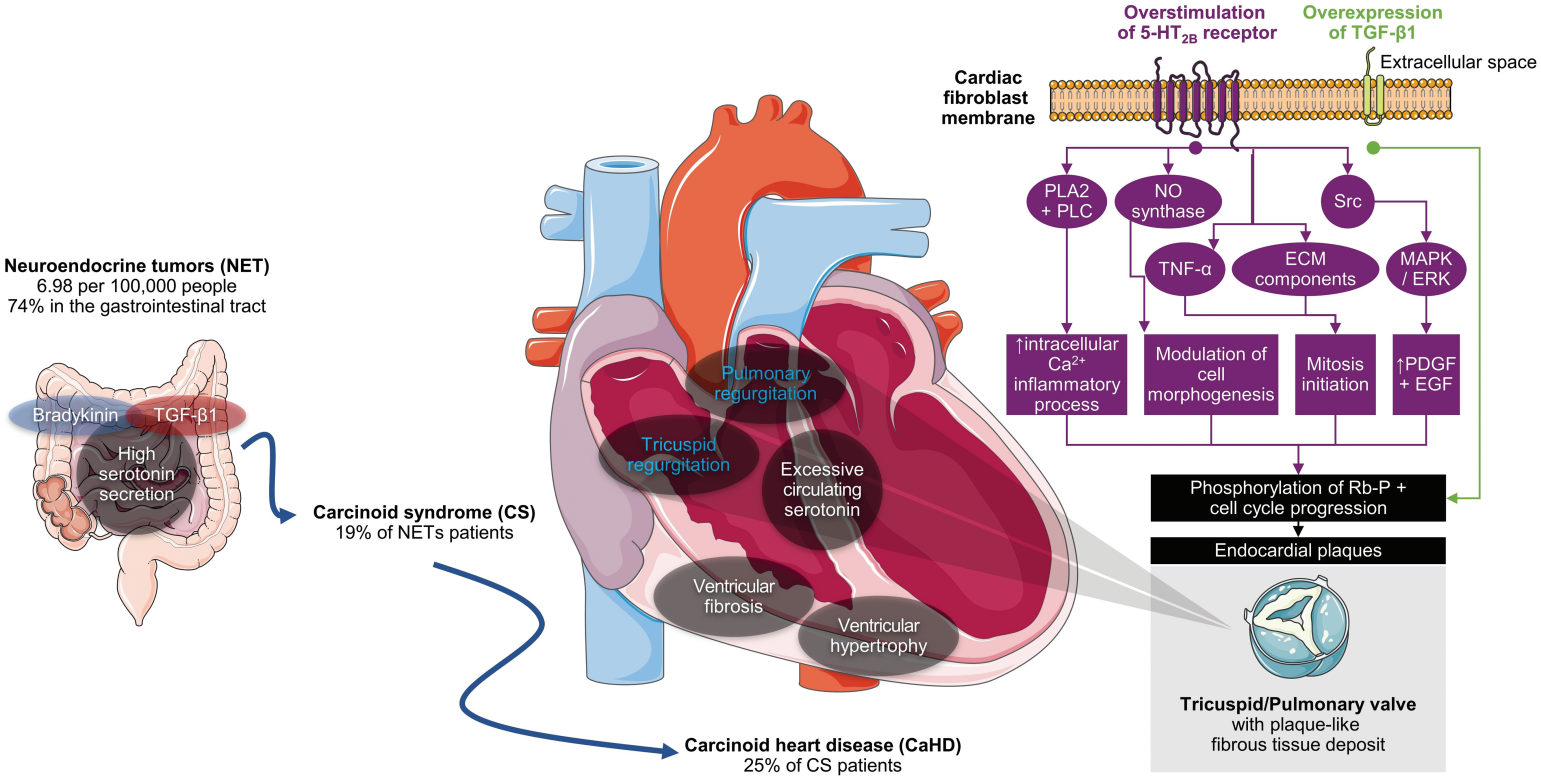
Effects of excess serotonin and other factors on valve fibrosis in CaHD.^5,7,8,15,41,59,60^ Ca^2+^, calcium ion; ECM, extracellular matrix; ERK, extracellular-regulated kinase; MAPK, mitogen-activated protein kinase; NO, nitric oxide; PLA2, phospholipase A2; PLC, phospholipase C; TGF, transforming growth factor; TNF-α, tumor necrosis factor alpha. Adapted from Servier Medical Art by Servier is licensed under a Creative Commons Attribution 3.0 unported license. Available at smart.servier.com. Accessed July 27, 2022.

Carcinoid heart disease (CaHD) is defined by characteristic plaque-like fibrous tissue deposits composed of myofibroblasts, smooth muscle cells, and extracellular matrix enveloped in an endothelial layer (Fig. 1).^1,2^ Of the approximately 180 000 patients with neuroendocrine tumors (NETs) in the US, 19% develop carcinoid syndrome (CS); among the patients who develop CS, 25%-65% may develop CaHD.^3-8^ The exact estimates of CaHD incidence/prevalence are limited by the methodology of interrogating studies. Although the pathophysiology behind CaHD is not clearly defined, the majority of relevant published literature attributes its core mechanism to chronic overexposure to serotonin in cardiac cells.^9^ Serotonin potentiates mitosis in fibroblasts and smooth muscle cells and can induce cardiac fibrosis and ­valvulopathy.^9-12^ Along with other factors (eg, substance P, neuropeptide K, transforming growth factor-β), serotonin contributes to plaque deposition on various endocardial surfaces such as the valve leaflets, subvalvular apparatus, and cardiac chambers.^13-15^ Symptoms arising from CaHD include congestive heart failure associated with valvular dysfunction (most commonly involving the tricuspid and pulmonic valves), dyspnea, fatigue, ascites, peripheral edema, and pleural effusion^16,17^

Diagnosis of CaHD is challenging, as many patients remain initially asymptomatic for years before right heart failure symptoms arise at a mean age of 59 years.^18,19^ Early screening upon development of CS and prior to appearance of CaHD symptoms is paramount, with either urinary or plasma 5-hydroxyindoleacetic acid (5-HIAA) levels and/or serum N-terminal pro B-type natriuretic peptide (NT-proBNP) ­levels.^6,20-22^ After a positive screening test, transthoracic 2-dimensional echocardiography is the most widely accepted initial imaging tool for both diagnosis and monitoring of CaHD.^6^ Echocardiographic findings in patients with CaHD demonstrate right-sided disease with tricuspid regurgitation is evident in all patients due primarily to thickened, retracted, immobile valve leaflets.^3,18,23^ Concurrent pulmonary regurgitation has been reported in 59%-88% of cases, with stenosis of either the tricuspid or pulmonary valve less commonly reported in approximately one-third of patients with CaHD.^3,18^ Left-sided disease is involved in <10% of cases, with regurgitation of the aortic and mitral valves prominent upon disease progression.^3,24^ CaHD diagnosis may be delayed 1.5 years or more, particularly if echocardiographic screening is not performed in patients with CS.^19^ Individual clinical practices vary, as echocardiographic screening is sometimes not done until patients present with symptoms—but by then, the disease has already advanced. The aim of this article is to review major CaHD guidance papers published prior to May 2022 and provide expert clinical perspectives on how to implement these recommendations into clinical practice, with the goal of increasing screening for CaHD, optimizing multidisciplinary collaboration, and discussing exploratory therapies to prevent the development of CaHD.

## Burden of CaHD

Right heart failure is inevitable for patients if their CaHD is not appropriately managed, resulting in a poor prognosis due to cardiac decompensation in up to 43% of patients and less than 1-year survival in many patients with unrepaired valvular disease.^15,24-26^ Compared to patients with CS without CaHD, the 3-year survival was consistently worse (31% vs. 68%) and mortality rates higher (27.8% vs. 13.0%).^18,27^ Patients who undergo tricuspid valve replacement have a 30-day mortality risk of 9%, and those surviving long-term have an improved prognosis of nearly 5 years and the added benefit of symptomatic relief.^28,29^

CaHD also poses an economic impacts for patients.^30^ Hospital admissions, length of stay, and outpatient services (eg, echocardiography) were the drivers of healthcare resource use and costs among patients receiving somatostatin analogs (SSA) for CS with CaHD compared to those without CaHD.^30,31^ Total average healthcare costs were $51,825 among patients with CaHD vs $29,068 for those without.^30^

## Biomarkers of CaHD

### Urinary/Plasma 5-HIAA

When carcinoid tumors metastasize to the liver or develop substantial disease bulk, the tumor can secrete excessive levels of serotonin, which is later metabolized to 5-HIAA.^32^ Levels of 5-HIAA are 2-4 times higher among patients with CaHD than in NET patients without CaHD, as higher risk for CaHD correlates with higher 5-HIAA levels.^4,33^ The threshold for elevated urinary 5-HIAA levels has been set at >300 μmol/24 h (57 mg/24 h) at screening across multiple guidance papers based on data that shows a 2.7-fold higher risk for CaHD with 5-HIAA levels 300-599 μmol/24 h (57-113 mg/24 h), 3.2-fold higher risk for CaHD with 5-HIAA levels 600-899 μmol/24 h (114-170 mg/24 h), and 3.4-fold higher risk for CaHD with 5-HIAA levels ≥900 μmol/24 h (≥171 mg/24 h).^4,6,21^ Patients who have been diagnosed with CaHD have ≥2-fold higher 5-HIAA levels than patients without CaHD, as mean 5-HIAA levels were reported at 556 µmol/24 h (106 mg/24 h) for mild disease, 408 µmol/24 h (78 mg/24 h) for moderate disease, and severe 901 µmol/24 h (171 mg/24 h) for severe disease.^34-36^ High 5-HIAA levels with a median range of 791-2247 µmol/24 h (150-442 mg/24 h) have been reported for disease progression and worsening of CaHD.^4,34,36,37^

The 300 µmol/24 h (57 mg/24 h) threshold for CaHD screening was based on urinary 5-HIAA levels; the appropriate threshold for plasma 5-HIAA has yet to be established as further research needs to be completed to determine appropriate cutoffs for patients at risk for CaHD.^4,6,21^ There exists a high concordance between plasma and urinary 5-HIAA, with precision varying only 3%-5% for within-day and ­day-to-day analyses of plasma and urinary 5-HIAA.^38,39^ Plasma 5-HIAA offers the convenience and reproducibility of a single blood sample rather than a 24-h urine collection that requires prolonged dietary restriction to avoid serotonin-rich food.^40^ Disease progression can be monitored with repeated urinary or plasma 5-HIAA levels, as evaluating trends over time may be more informative than a single measurement in time.^22^ Elevated 5-HIAA levels correlate with worsening CaHD, as studies have shown these patients to have >25% cardiac score elevation, with every 100 nmol/L increase in plasma 5-HIAA, yielding a 5% greater odds of disease progression.^26,37^ High 5-HIAA levels also correlate with risk of death due to CaHD, as every 52 µmol/24 h (10 mg/24 h) increase in urinary 5-HIAA correlates with an 11.8% increase in 1-year mortality.^41^

### Serum NT-proBNP

NT-proBNP is an inactive neurohormone released in response to high ventricular wall stress due to cardiac volume and pressure overload.^42^ Baseline NT-proBNP assessment is recommended at the time of CS diagnosis.^21^ Across multiple studies, the median NT-proBNP levels were higher among patients with CaHD compared to those without CaHD.^3,43^ Higher NT-proBNP levels correlate with more symptoms and worse functional NYHA class.^43^ Serum NT-proBNP levels were found to be elevated among patients with high risk of developing CaHD (even prior to onset of symptoms) compared to NET patients who are at low risk for developing CaHD.^44^ NT-proBNP levels are a reflection of the consequences of CaHD, not just a predictor of patients at risk for CaHD.^44^ NT-proBNP has been suggested as a reasonable early diagnostic marker of CaHD, as it appears to be produced by cardiomyocytes when under mild or moderate strain even before an echocardiographic finding of CaHD can be detected.^44^ NT-proBNP levels also correlate with worsening CaHD, as patients with levels <55 pg/mL (<6.5 pmol/L) indicate a need for active NT-proBNP screening but not necessarily for echocardiographic screening, whereas patients with levels >55 pg/mL (<6.5 pmol/L) would benefit from echocardiography once or twice annually, and patients with levels >226 pg/mL (>27 pmol/L) would benefit from echocardiography every 6 months.^44^

The ACC and ENETS guidance papers have suggested a threshold of >260 pg/mL (31 pmol/L) for NT-proBNP as a biomarker for CaHD.^6,21^ With an NT-proBNP threshold of >260 pg/mL (31 pmol/L) to refer patients for echocardiography, only 1.4 patients need to undergo echocardiography to detect 1 patient with CaHD.^6,21,43^ Echocardiogram reports should address the status of leaflet thickening, mobility, degree of regurgitation, and stenosis of each affected valve and assess the right ventricular size and function.^21^ A positive correlation exists between NT-proBNP and severity of symptoms for CS, and overall mortality.^43,45^ NT-proBNP is considered to be the most useful diagnostic tool, predictor of CaHD, and adjunct marker validated with echocardiographic findings^21,42^ NT-proBNP is generally accessible due to its ease of use and relatively low cost, however, certain medical centers restrict its ordering for only patients on specific heart failure medications. Although NT-proBNP was originally identified as a cardiac biomarker, its utility is now expanded as a ­cardio-oncology biomarker.^46^

### Other Biomarkers

Chromogranin A (CgA), a glycoprotein produced by NET cells, is a non-specific biomarker for severe CaHD.^6^ CgA is not routinely used clinically by most NET specialists to guide NET therapy.^20^ In addition, CgA is also not routinely recommended for CaHD surveillance by any of the assessed guideline papers. Its metabolite, pancreastatin, may be of more utility in predicting survival since it is unaffected by proton pump inhibitor use and pernicious anemia, unlike CgA.^47^ Activin A, like CgA, was found to have high sensitivity but low specificity for CaHD.^48^ The role of other biomarkers with potentially better diagnostic utility such as the 51-gene, polymerase chain reaction-based NETest are still under investigation.^49^

## Screening

The North American Neuroendocrine Tumor Society (NANETS) Consensus Guidelines, ACC Expert Statement, and ENETS Guidance Paper are collectively referred to as guidance papers for the purposes of this review. Most of these guidance papers provided by leading organizations for the management of CaHD focus on the importance of early screening with 5-HIAA, NT-proBNP levels, and echocardiograms (Table 1).^6,20,21^ Ultimately, the goal is to identify patients who are truly at high risk of CaHD using either urinary or plasma 5-HIAA or NT-proBNP levels and follow these patients with echocardiographic surveillance at an appropriate frequency to capture the risk of developing CaHD.

**Table 1.** Comparison of select CaHD/CS evaluation recommendations from major guidance papers.

	NCCN^22^	NANETS^20^	ACC^6^	ENETS^21^
5-HIAA	Urinary or plasma 5-HIAA	Threshold of >5× ULN urinary or plasma 5-HIAA to undergo annual echocardiogram	>300 µmoL/24 h urinary or plasma levels; screen all patients with small intestinal NETs	≥300 µmol/24 h urinary or plasma
NT-proBNP	—	Negative predictive value	>260 pg/mL or 31 pmol/L; screen every 6 months for CaHD in patients with CS	>260 pg/mL or 31 pmol/L; screen patients with ↑ 5-HIAA or symptoms at baseline and follow-up
Echocardiogram	TTE every 1-3 years or as clinically indicated for morphologic evaluation of the valves (especially tricuspid and pulmonic valves) and right heart	Annual echocardiogram in patients with ↑ serotonin or 5-HIAA	TTE every 3-6 months for patients with CS and high suspicion of CaHD; 3D TTE preferred over 2D TTE to assess valve pathology	TTE every 6-12 months to screen patients with ↑ NT-proBNP
Other imaging	^68^Ga DOTATATE or ^68^Ga DOTATOC or ^64^Cu DOTATATE PET scan to assess somatostatin receptor status	^68^Ga DOTATATE PET scan for overall tumor assessment	CMR scans valuable as adjuncts; CCT accurate for left/right ventricular volume calculations	CMR scans and CCT useful as alternatives

Abbreviations: 2D, 2-dimensional; 3D, 3-dimensional; 5-HIAA, 5-hydroxyindoleacetic acid; ^68^Ga, radioactive gallium 68; ACC, American College of Cardiology; CaHD, carcinoid heart disease; CCT, cardiac computed tomography; CMR, cardiac magnetic resonance; CS, carcinoid syndrome; ENETS, European Neuroendocrine Tumor Society; NANETS, North American Neuroendocrine Tumor Society; NCCN, National Comprehensive Cancer Network ; NET, neuroendocrine tumor; NT-proBNP, N-terminal pro B-type natriuretic peptide; TTE, transthoracic echocardiography; ULN, upper limit of normal.

ENETS and ACC agree on 300 µmol/24 h (57 mg/24 h) as the 5-HIAA threshold for CaHD screening, with NANETs being an outlier by setting a relative threshold as 5× the upper limit of normal (ULN) for 5-HIAA levels.^6,20,21^ NCCN Clinical Practice Guidelines in Oncology (NCCN Guidelines) do not endorse a specific threshold but it does cite the same study which establishes an increased risk of developing CaHD in CS patients with 5-HIAA levels ≥300 µmol/24 h (57 mg/24 h).^4,22^

ACC and ENETS designate 260 pg/mL (31 pmol/L) as the NT-proBNP threshold for CaHD screening, while ACC and NANETS recognize its negative predictive value and NCCN Guidelines does not specifically comment on threshold ­levels.^6,20-22^ ACC cites a study with 200 CS patients showing significantly higher NT-proBNP levels among patients with CaHD compared to those without CaHD (median 1149 pg/mL vs. 101 pg/mL, respectively; *P* < .001), and a negative predictive value of 98%.^6,43^ The high negative predictive value reflects the proportion of negative results that are true negatives—the likelihood that individuals with low NT-proBNP levels do not have CaHD.^50^

The recommended frequency of echocardiograms varies widely among the guidance papers, as ACC suggests the most aggressive monitoring with TTE every 3-6 months compared to less frequently: 6-12 months by ENETS, annually by NANETS, and every 1-3 years or as clinically indicated by NCCN Guidelines.^6,20-22^ Other imaging modalities such as the cardiac magnetic resonance (CMR) scanning and cardiac computed tomography (CCT) are emerging as useful tools for surgical planning to visualize right-sided heart function often not well characterized using echocardiograms (Table 1).^6,21^ For pulmonary valve evaluation, ACC specifically advocates CMR since the structure of the pulmonary valve is otherwise difficult to visualize.^21,51^ Somatostatin-receptor imaging using radiopeptide gallium 68 (^68^Ga) DOTATATE or ^68^Ga DOTATOC or copper 64 (^64^Cu) DOTATATE positron emission photography (PET) scan is appropriate for overall tumor assessment^20,22^ (Table 1).

Other screening measures include physical exams and electrocardiograms (EKGs). Clinical diagnosis of CaHD is not straightforward as patients with even moderate or severe tricuspid regurgitation are clinically asymptomatic (no cardiac murmur, elevated jugular venous pressure, and lower extremity edema) in up to 60% of cases.^52^ EKGs also carry limited screening potential in CaHD with patients in later stages of CaHD demonstrating non-specific low QRS voltage.^6^

## Management Options

The overriding goal of therapy for a patient with CaHD is the aggressive reduction of NET hormone secretion while managing complications arising from right heart failure.^21^ Across the 4 major guidance papers for CaHD/CS, all recommend the use of SSAs as first-line anti-tumor therapy and as means of controlling carcinoid syndrome (Table 2).^6,20-22^ Based on a recent meta-analysis of 1945 patients receiving SSA across 33 studies, it was determined that all formulations (short-acting or long-acting) of either octreotide or lanreotide were equally efficacious in reducing diarrhea and flushing symptoms in 66%-70% and reducing 5-HIAA levels in 45%-46% of patients (Hofland et al 2019). By escalating the dose (for long-acting octreotide) or decreasing the injection dosing interval to 21 days (for long-acting octreotide or lanreotide), SSA therapy can be optimized to further reduce diarrhea symptoms by 72% and flushing symptoms by 84%.^53^ However, dose escalation of the SSA only appears to reduce 5-HIAA levels further in 29% of patients.^53^

**Table 2.** Comparison of select CaHD/CS management recommendations from major guidance papers.

	NCCN^22^	NANETS^20^	ACC^6^	ENETS^21^
Anti-tumor therapies/control of CS syndrome	1st line: SSA^a^ 2nd line: depending on site of disease, disease stage, line of therapy, and other factors, treatment options may include PRRT^b^, systemic therapy, surgical cytoreduction, hepatic arterial embolization	1st line: SSA^a^ 2nd line: PRRT^b^, everolimus, liver embolization Last: IFN-α	1st line: SSA^a^ 2nd line: IFN-α, PRRT^c^ Use with caution: everolimus, TAE, surgical hepatic debulking	1st line: SSA^a^ 2nd line: PRRT^b^, everolimus, IFN-α Use with caution: surgical hepatic resection, TAE, TACE, TARE/SIRT
Diarrhea volume control^d^	Telotristat ethyl	Telotristat ethyl	Telotristat ethyl	Telotristat ethyl
HF symptom control	—	—	Loop and thiazide diuretics, aldosterone	Loop and thiazide diuretics, aldosterone
Embolization therapy	—	Bland embolization, chemoembolization, and radioembolization can be appropriate		
Valve replacement surgery	Valve replacement (typically tricuspid and pulmonary) indicated for patients with symptomatic disease	Bioprosthetic preferred over mechanical tricuspid and pulmonary valves for moderate-severe CaHD with life expectancy >1 year	Bioprosthetic preferred over mechanical valve; percutaneous catheter-based interventions for high-risk patients	Bioprosthetic preferred over mechanical valve; percutaneous valve-in-valve in degenerated surgical bioprosthesis

^a^Octreotide LAR and lanreotide.

^b177^Lu-DOTATATE (if SSTR-positive and progression on octreotide LAR/lanreotide).

^c^Yttrium Y 90-edotreotide or luteium-DOTATATE.

^d^Refractory to SSA.

Abbreviations: ACC, American College of Cardiology; CaHD, carcinoid heart disease; CS, carcinoid syndrome; CS, carcinoid syndrome; ENETS, European Neuroendocrine Tumor Society; HF, heart failure; IFN-α, interferon alpha; LAR, long-acting repeatable; NANETS, North American Neuroendocrine Tumor Society; NCCN, National Comprehensive Cancer Network ; PRRT, peptide receptor radiotherapy; SSA, somatostatin analogs; TACE, trans-arterial chemoembolization; TAE, transcatheter arterial embolization; TARE/SIRT, trans-arterial radioembolization/selective internal radiation therapy.

For diarrhea volume not controlled by SSA alone, telotristat is consistently recommended by all 4 guidance papers to lower serotonin/5-HIAA levels as it has been shown to achieve a diarrhea-reducing response in 40%-44% of refractory patients (Table 2).^6,20-22,53-55^

Loop and thiazide diuretics, along with aldosterone, are the mainstay of treatment for heart failure symptoms from CaHD based on ACC and ENETS (Table 2).^6,21^ Tricuspid with or without pulmonic bioprosthetic valve replacement is preferred over mechanical valve replacement for patients with severe CaHD and ≥12 months of anticipated post-operative survival to avoid long term anticoagulation in these patients who may patients with CaHD and CS who may have a propensity for bleeding (Table 2).^6,20,21^ Percutaneous interventions are reserved for selected patients (Table 2).^6,21^

## Gaps in Management Options

Despite numerous consensus statements and guidelines from national and international societies screening, diagnosis, and treatment practices differ and are not uniform in patients with CaHD. The sequence of post-SSA therapies for patients with CaHD is not well-defined based on current guidance papers, but the options generally include PRRT, everolimus, or liver embolization (Table 2).^6,20-22^ Selection of a second-line treatment depends on multiple factors, including site of primary tumor, disease bulk, patient co-morbidities, and tumor functional status. If liver disease is predominant, then liver-directed therapies such as debulking or embolization may be considered above others. Systemic PRRT achieves symptomatic response in 64%-74% of patients, but biochemical response in only 17% of patients.^52^ Recent studies have also suggested that earlier incorporation of PRRT post-SSA may increase the anti-tumor benefit from the therapy patients derive.^56^ The addition of everolimus to SSA therapy improves biochemical response among 61% of patients, incrementally better than the biochemical response rate of 54% among patients receiving SSA monotherapy.^53^ The various liver embolization therapies generally achieve symptom reduction and biochemical response in 82% and 63% of patients, respectively.^53^

Other challenges associated with CaHD include appropriate screening to diagnose CaHD as early as possible with consistent biomarker and echocardiographic screening and more collaboration between cardiologists and oncologists for interdisciplinary management of CaHD.^21^ Oncologists can collaborate with cardiologists by referring patients with early CaHD for additional testing, leveraging the expertise and resources of heart failure specialists and oncology-focused cardiology programs emerging at major academic centers. In certain medical centers, the ability to order NT-proBNP levels may be restricted to cardiologists—making it necessary for the oncologist to initiate a cardiology referral for simple screening; improving accessibility to NT-proBNP screening would facilitate the more efficient assessment of CaHD risk. Cardiologists are otherwise typically not consulted for CaHD until valvular dysfunction is evident, or when other cardiac issues (eg, arrhythmias, tachycardia, blood pressure) arise that require a cardiology work-up which may delay appropriate treatment. Once oncologists identify patients at high risk for CaHD, baseline 5-HIAA levels, NT-proBNP levels, and echocardiographic findings would be valuable data points for cardiologists who follow the patients through the progression of the disease. Cardiothoracic surgery or interventional radiology consultation may also be warranted to explore treatment options beyond systemic therapy (eg, embolization, debulking). With early integration of care for patients with symptoms, other cardiac sequelae can be detected by specialists and potentially result in better outcomes.

Although guidance papers exist for the appropriate management of CaHD, execution often falls short in clinical ­practice.^6,20-22,57^ Recent reports indicate that <42% of patients who meet at least 1 criterion for CaHD screening actually receive an echocardiogram—indicating an opportunity to improve screening rates.^57^The best ways to improve the adoption of these strategies is through ongoing education and collaboration between cardiologists, cardiothoracic surgeons/interventional radiologists, and oncologists.

There are 2 studies underway to evaluate treatment options for patients with CaHD. One involves telotristat ethyl in a study called TELEHEART (TELotristat Ethyl in a HEART biomarker study; ClinicalTrials.gov Identifier NCT04810091) and the other involves lutetium 177 (^177^Lu) DOTATATE in a study called CHARRT (Carcinoid Heart disease And peptide Receptor Radiotargetted Therapy; ClinicalTrials.gov Identifier NCT04039516). The TELEHEART study is a randomized, double-blind, placebo-controlled, ­single-center phase III study in which patients with metastatic and/or locally advanced NETs on SSA therapy with CS or CS and CaHD are randomized to 6 months of telotristat plus SSA or SSA alone. This study is currently recruiting. The efficacy assessments are based on NT-proBNP response, 5-HIAA levels, a 6-min walk test, and echocardiographic parameters over 6 months (ClinicalTrials.gov Identifier NCT04810091). Early preclinical data suggests telotristat may be able to reverse myxomatous valvular degeneration in mouse models of CaHD, however, it remains to be seen whether this mechanism can translate to human patients.^58^ CHARRT is a randomized, open-label, multicenter phase II study in which patients with stable mild/moderate CaHD and CS are assigned in a parallel fashion to PRRT with ^177^Lu DOTATATE plus SSA therapy or SSA therapy alone. This study has not yet started enrollment. The endpoints of the study include evaluating the progression of moderate CaHD based on response evaluation criteria in solid tumors (RECIST) computed tomography/magnetic resonance imaging, urinary 5-HIAA levels, and New York Heart Association (NYHA) Heart Failure Score over 5 years (ClinicalTrials.gov Identifier NCT04039516). When the results of the TELEHEART and CHARRT studies become available, we will be able to further determine if changes in NT-proBNP and echocardiographic parameters are meaningful surrogate endpoints in CaHD, and if therapeutic interventions are able to modify the disease biology of CaHD.

There is an unmet need for effective strategies to prevent the occurrence of CaHD. Since there is an association between CaHD and elevated serum serotonin, reductions in serotonin levels should theoretically reduce the risk of CaHD development.^20^ As there are yet to be any means of reversing CaHD, it would be ideal to be able to prevent the disease from happening rather than simply react to the consequences of CaHD after has taken root. NANETs suggest consideration of telotristat therapy for patients with significantly high urinary 5-HIAA levels and signs of valvular damage on echocardiogram.^20^

## Strategies to Screen, Diagnose, and Manage CaHD

All NET patients with CS symptoms should have levels of either urinary or plasma 5-HIAA and NT-proBNP screened at baseline and, at minimum, every 6 months. If the level of either 5-HIAA is ≥300 µmol/24 h (≥57 mg/24 h) or NT-proBNP is >260 pg/mL (31 pmol/L), an echocardiogram should be evaluated and obtained along with a cardiology consult. If no evidence of CaHD is found, repeat echocardiography could be performed every 3-6 months. Diagnosis of CaHD is generally made based on echocardiogram findings.

Optimization of existing SSA therapy may help reduce CS symptoms. Adding telotristat should be considered if better control of diarrhea is needed. Among patients who have already developed heart failure symptoms, diuretics should be initiated while valve replacement is considered. Debulking or embolization is preferred if liver disease is prominent. Otherwise, systemic PRRT or everolimus is useful to control symptoms.

## Conclusion

The ENETS and ACC guidance papers are the most aligned in their recommendations for screening for the development of CaHD in patients. Disparities with the NCCN Guidelines and NANETS guidance documents may be byproducts of the compositions of the panels (by specialty groups represented) and lack of availability of data. The nuances between these (NCCN Guidelines, NANETs, ACC, and ENETS) may confuse healthcare providers, however, we are optimistic that these issues can be resolved by including the perspectives of both the cardiologists and the oncologists. As the authorship of this article represents the collaboration between both specialties, our expert opinion provides some clarification through review of recent data, clinical perspectives, and recommendations for future research in this disease space. Early screening with 5-HIAA, NT-proBNP levels, and echocardiography upon recognition of CS and prior to the development of CaHD symptoms is paramount. Multidisciplinary collaboration would be beneficial for management of CaHD patients with CaHD as well as for designing studies focused on preventing the onset of CaHD in at-risk patients with CS.

## Data Availability

No new data were generated or analyzed in support of this research.

## References

[1] Ferrans VJ , Roberts WC. The carcinoid endocardial plaque; an ultrastructural study. Hum Pathol. 1976;7(4):387-409. 10.1016/s0046-8177(76)80054-8. 939537

[2] Bernheim AM , Connolly HM, Hobday TJ, Abel MD, Pellikka PA. Carcinoid heart disease. Prog Cardiovasc :Dis. 2007;49(6):439-451. 10.1016/j.pcad.2006.12.002. 17498524

[3] Baron E , Szymanski C, Hergault H, et al. Progression of carcinoid heart disease in the modern management era. J Am Heart Assoc. 2021;10(23):e020475. 10.1161/JAHA.120.020475. PMC9075379 34816734

[4] Bhattacharyya S , Toumpanakis C, Chilkunda D, Caplin ME, Davar J. Risk factors for the development and progression of carcinoid heart disease. Am J Cardiol. 2011;107(8):1221-1226. 10.1016/j.amjcard.2010.12.025. 21296329

[5] Dasari A , Shen C, Halperin D, et al. Trends in the incidence, prevalence, and survival outcomes in patients with neuroendocrine tumors in the United States. JAMA Oncol. 2017;3(10):1335-1342. 10.1001/jamaoncol.2017.0589. 28448665 PMC5824320

[6] Davar J , Connolly HM, Caplin ME, et al. Diagnosing and managing carcinoid heart disease in patients with neuroendocrine tumors: an expert statement. J Am Coll Cardiol. 2017;69(10):1288-1304. 10.1016/j.jacc.2016.12.030. 28279296

[7] Halperin DM , Shen C, Dasari A, et al. Frequency of carcinoid syndrome at neuroendocrine tumour diagnosis: a population-based study. Lancet Oncol. 2017;18(4):525-534. 10.1016/s1470-2045(17)30110-9. 28238592 PMC6066284

[8] Macfie R , McCully BH, Ratzlaff AN, et al. The prevalence, operations, and outcomes of carcinoid heart disease. Am J Surg. 2022;224(2):665-669. 10.1016/j.amjsurg.2022.03.054 35382934

[9] Laskaratos F-M , Rombouts K, Caplin M, et al. Neuroendocrine tumors and fibrosis: an unsolved mystery?. Cancer. 2017;123(24):4770-4790. 10.1002/cncr.31079. 29112233

[10] Seuwen K , Magnaldo I, Pouysségur J. Serotonin stimulates DNA synthesis in fibroblasts acting through 5-HT1B receptors coupled to a Gi-protein. Nature. 1988;335(6187):254-256. 10.1038/335254a0. 3045568

[11] Lee SL , Wang WW, Lanzillo JJ, Fanburg BL. Serotonin produces both hyperplasia and hypertrophy of bovine pulmonary artery smooth muscle cells in culture. Am J Physiol. 1994;266(1 PT 1):46-52. 10.1152/ajplung.1994.266.1.L46. 8304469

[12] Gustafsson BI , Tømmerås K, Nordrum I, et al. Long-term serotonin administration induces heart valve disease in rats. Circulation. 2005;111(12):1517-1522. 10.1161/01.CIR.0000159356.42064.48. 15781732

[13] Lundin L , Norheim I, Landelius J, Oberg K, Theodorsson-Norheim E. Carcinoid heart disease: relationship of circulating vasoactive substances to ultrasound-detectable cardiac abnormalities. Circulation. 1988;77(2):264-269. 10.1161/01.cir.77.2.264. 2448062

[14] Waltenberger J , Lundin L, Oberg K, et al. Involvement of transforming growth factor-beta in the formation of fibrotic lesions in carcinoid heart disease. Am J Pathol. 1993;142(1):71-78. 8424467 PMC1886850

[15] Grozinsky-Glasberg S , Grossman AB, Gross DJ. Carcinoid heart disease: from pathophysiology to treatment—“Something in the way it moves.”. Neuroendocrinology. 2015;101(4):263-273. 10.1159/000381930. 25871411

[16] Pandya UH , Pellikka PA, Enriquez-Sarano M, et al. Metastatic carcinoid tumor to the heart: echocardiographic-pathologic study of 11 patients. J Am Coll Cardiol. 2002;40(7):1328-1332. 10.1016/s0735-1097(02)02109-5. 12383582

[17] Bober B , Saracyn M, Kołodziej M, et al. Carcinoid heart disease: how to diagnose and treat in 2020?. Clin Med Insights Cardiol. 2020;14:1179. 10.1177/1179546820968101. PMC7597558 33192110

[18] Pellikka PA , Tajik AJ, Khandheria BK, et al. Carcinoid heart disease. Clinical and echocardiographic spectrum in 74 patients. Circulation. 1993;87(4):1188-1196. 10.1161/01.cir.87.4.1188. 7681733

[19] Møller JE , Pellikka PA, Bernheim AM, et al. Prognosis of carcinoid heart disease: analysis of 200 cases over two decades. Circulation. 2005;112(21):3320-3327. 10.1161/circulationaha.105.553750. 16286584

[20] Strosberg JR , Halfdanarson TR, Bellizzi AM, et al. The North American Neuroendocrine Tumor Society consensus guidelines for surveillance and medical management of midgut neuroendocrine tumors. Pancreas. 2017;46(6):707-714. 10.1097/mpa.0000000000000850. 28609356 PMC5642985

[21] Grozinsky-Glasberg S , Davar J, Hofland J, et al. European Neuroendocrine Tumor Society (ENETS) 2022 Guidance Paper for Carcinoid Syndrome and Carcinoid Heart Disease. J Neuroendocrinol. 2022;34(7):ee13146. 10.1111/jne.13146. PMC9539661 35613326

[22] .Referenced with permission from the NCCN Clinical Practice Guidelines in Oncology (NCCN Guidelines®) for Neuroendocrine and adrenal tumors V.1.2022. © National Comprehensive Cancer Network, Inc. All rights reserved. Accessed June 16, 2022. To view the most recent and complete version of the guideline, go online to NCCN.org. NCCN makes no warranties of any kind whatsoever regarding their content, use or application and disclaims any responsibility for their application or use in any way.

[23] Fijalkowski R , Reher D, Rinke A, et al. Clinical features and prognosis of patients with carcinoid syndrome and carcinoid heart disease: a retrospective multicentric study of 276 patients. Neuroendocrinology. 2022;112(6):547-554. 10.1159/000518651. 34348326

[24] Jin C , Sharma AN, Thevakumar B, et al. Carcinoid heart disease: pathophysiology, pathology, clinical manifestations, and management. Cardiology. 2021;146(1):65-73. 10.1159/000507847. 33070143

[25] Ross EM , Roberts WC. The carcinoid syndrome: comparison of 21 necropsy subjects with carcinoid heart disease to 15 necropsy subjects without carcinoid heart disease. Am J Med. 1985;79(3):339-354. 10.1016/0002-9343(85)90313-4. 4036985

[26] Dobson R , Burgess MI, Valle JW, et al. Serial surveillance of carcinoid heart disease: factors associated with echocardiographic progression and mortality. Br J Cancer. 2014;111(9):1703-1709. 10.1038/bjc.2014.468. 25211656 PMC4453728

[27] Bergestuen DS , Aabakken L, Holm K, Vatn M, Thiis-Evensen E. Small intestinal neuroendocrine tumors: prognostic factors and survival. Scand J Gastroenterol. 2009;44(9):1084-1091. 10.1080/00365520903082432. 19572232

[28] .Nguyen A, Schaff HV, Abel MD, Luis SA, Lahr BD, Halfdanarson TR, Connolly HM. Improving outcome of valve replacement for carcinoid heart disease. J Thorac Cardiovasc Surg. 2019;158:99-107.e2. 10.1016/j.jtcvs.2018.09.025 30527716

[29] O’Malley TJ , Jimenez DC, Saxena A, et al. Outcomes of surgical treatment for carcinoid heart disease: a systematic review and meta-analysis. Surgery. 2021;170(2):390-396. 10.1016/j.surg.2021.02.054. 33812754

[30] .Joish VN, Perez-Olle R, Lapuerta P, Dharba S, Zacks J. Burden of carcinoid heart disease in patients with carcinoid syndrome initiating somatostatin analogues. Clin Ther. 2019;41:1716-1723.e2. 10.1016/j.clinthera.2019.06.013 31326125

[31] Lesén E , Björstad A, Björholt I, et al. Real-world treatment patterns, resource use and costs of treating uncontrolled carcinoid syndrome and carcinoid heart disease: a retrospective Swedish study. Scand J Gastroenterol. 2018;53(12):1509-1518. 10.1080/00365521.2018.1531653. 30449217

[32] Feldman JM. Serotonin metabolism in patients with carcinoid tumors: incidence of 5-hydroxytryptophan-secreting tumors. Gastroenterology. 1978;75(6):1109-1114. 10.1016/0016-5085(78)90084-7. 309417

[33] Zuetenhorst JM , Bonfrer JMGM, Korse CM, et al. Carcinoid heart disease: the role of urinary 5-hydroxyindoleacetic acid excretion and plasma levels of atrial natriuretic peptide, transforming growth factor-beta and fibroblast growth factor. Cancer. 2003;97(7):1609-1615. 10.1002/cncr.11226. 12655516

[34] Denney WD , Kemp WE Jr, Anthony LB, Oates JA, Byrd BF 3rd. Echocardiographic and biochemical evaluation of the development and progression of carcinoid heart disease. J Am Coll Cardiol. 1998;32(4):1017-1022. 10.1016/s0735-1097(98)00354-4. 9768727

[35] Westberg G , Wängberg B, Ahlman H, et al. Prediction of prognosis by echocardiography in patients with midgut carcinoid syndrome. Br J Surg. 2001;88(6):865-872. 10.1046/j.0007-1323.2001.01798.x. 11412260

[36] Buchanan-Hughes A , Pashley A, Feuilly M, et al. Carcinoid heart disease: prognostic value of 5-hydroxyindoleacetic acid levels and impact on survival: a systematic literature review. Neuroendocrinology. 2021;111(1-2):1-15. 10.1159/000506744. 32097914

[37] Møller JE , Connolly HM, Rubin J, et al. Factors associated with progression of carcinoid heart disease. N Engl J Med. 2003;348(11):1005-1015. 10.1056/NEJMoa021451. 12637610

[38] Pussard E , Guigueno N, Adam O, Giudicelli JF. Validation of HPLC-amperometric detection to measure serotonin in plasma, platelets, whole blood, and urine. Clin Chem. 1996;42(7):1086-1091. 10.1093/clinchem/42.7.1086. 8674193

[39] Adaway JE , Dobson R, Walsh J, Cuthbertson DJ, Monaghan PJ, Trainer PJ, Valle JW, Keevil BG. Serum and plasma ­5-hydroxyindoleacetic acid as an alternative to 24-h urine ­5-hydroxyindoleacetic acid measurement. Ann Clin Biochem. 2016;53(Pt 5):554-560. 10.1177/0004563215613109 26438520

[40] Wedin M , Mehta S, Angerås-Kraftling J, Wallin G, Kaskalakis K. The role of serum 5-HIAA as a predictor of progression and an alternative to 24-h urine 5-HIAA in well-differentiated neuroendocrine neoplasms. Biology (Basel). 2021;10(2):76. 10.3390/biology10020076. 33494283 PMC7909826

[41] Joish VN , Shah S, Tierce JC, et al. Serotonin levels and 1-year mortality in patients with neuroendocrine tumors: a systematic review and meta-analysis. Future Oncol. 2019;15(12):1397-1406. 10.2217/fon-2018-0960. 30734573

[42] Zuetenhorst JM , Korse CM, Bonfrer JMG, Bakker RH, Taal BG. Role of natriuretic peptides in the diagnosis and treatment of patients with carcinoid heart disease. Br J Cancer. 2004;90(11):2073-2079. 10.1038/sj.bjc.6601816. 15150565 PMC2409483

[43] Bhattacharyya S , Toumpanakis C, Caplin ME, Davar J. Usefulness of N-terminal pro-brain natriuretic peptide as a biomarker of the presence of carcinoid heart disease. Am J Cardiol. 2008;102(7):938-942. 10.1016/j.amjcard.2008.05.047. 18805126

[44] Levy S , Kilgallen AB, Korse CM, et al. Elevated serotonin and NT-proBNP levels predict and detect carcinoid heart disease in a large validation study. Cancers (Basel). 2022;14(10):2361. 10.3390/cancers14102361. 35625964 PMC9139809

[45] Korse CM , Taal BG, de Groot CA, Bakker RH, Bonfrer JMG. Chromogranin-A and N-terminal pro-brain natriuretic peptide: an excellent pair of biomarkers for diagnostics in patients with neuroendocrine tumor. J Clin Oncol. 2009;27(26):4293-4299. 10.1200/JCO.2008.18.7047. 19667278

[46] Chovanec J , Chovanec M, Mego M. Levels of NT-proBNP and troponin T in cancer patients—mini-review. Klin Onkol. 2020;33(3):171-176. 10.14735/amko2020171. 32683872

[47] Woltering EA , Beyer DT, Thiagarajan R, et al. Elevated plasma pancreastatin, but not chromogranin A, predicts survival in neuroendocrine tumors of the duodenum. J Am Coll Surg. 2016;222(4):534-542. 10.1016/j.jamcollsurg.2015.12.014. 26827125

[48] Bergestuen DS , Edvardsen T, Aakhus S, et al. Activin A in carcinoid heart disease: a possible role in diagnosis and pathogenesis. Neuroendocrinology. 2010;92(3):168-177. 10.1159/000318014. 20720391

[49] Malczewska A , Witkowska M, Wójcik-Giertuga M, et al. Prospective evaluation of the NETest as a liquid biopsy for gastroenteropancreatic and bronchopulmonary neuroendocrine tumors: an ENETS Center of Excellence experience. Neuroendocrinology. 2021;111(4):304-319. 10.1159/000508106. 32335553

[50] Monaghan TF , Rahman SN, Agudelo CW, et al. Foundational statistical principles in medical research: sensitivity, specificity, positive predictive value, and negative predictive value. Medicina (Kaunas). 2021;57(5):503. 10.3390/medicina57050503. 34065637 PMC8156826

[51] Bhattacharyya S , Toumpanakis C, Burke M, et al. Features of carcinoid heart disease identified by 2- and 3-dimensional echocardiography and cardiac MRI. Circ Cardiovasc Imaging. 2010;3(1):103-111. 10.1161/CIRCIMAGING.109.886846. 19920029

[52] Bhattacharyya S , Toumpanakis C, Caplin ME, Davar J. Analysis of 150 patients with carcinoid syndrome seen in a single year at one institution in the first decade of the twenty-first century. Am J Cardiol. 2007;101(3):378-381. 10.1016/j.amjcard.2007.08.045. 18237604

[53] Hofland J , Herrera-Martínez AD, Zandee WT, de Herder WW. Management of carcinoid syndrome: a systematic review and meta-analysis. Endocr Relat Cancer. 2019;26(3):R145-R156. 10.1530/ERC-18-0495. 30608900

[54] Kulke MH , Hörsch D, Caplin ME, et al. Telotristat ethyl, a tryptophan hydroxylase inhibitor for the treatment of carcinoid syndrome. J Clin Oncol. 2017;35(1):14-23. 10.1200/JCO.2016.69.2780. 27918724

[55] Pavel M , Gross DJ, Benavent M, et al. Telotristat ethyl in carcinoid syndrome: safety and efficacy in the TELECAST phase 3 trial. Endocr Relat Cancer. 2018;25(3):309-322. 10.1530/erc-17-0455. 29330194 PMC5811631

[56] Das S , Chauhan A, Du L, et al. External validation of a clinical score for patients with neuroendocrine tumors under consideration for peptide receptor radionuclide therapy. JAMA Netw Open. 2022;5(1):2144170. 10.1001/jamanetworkopen.2021.44170. PMC8771294 35044469

[57] Mouser B , Atari O, Howe J, O’Dorisio T, Parekh K, Bashir M. Screening for carcinoid heart disease: future perspectives. Poster Presented at: 71st Annual Scientific Session of American College of Cardiology; April 2-4, 2022; Washington, DC.

[58] Wang X , Kuban-Johnston D, Lapuerta P, Lacerda CMR. Telotristat ethyl reverses myxomatous changes in mice mitral valves. Front Cardiovasc Med. 2022;9:945672. 10.3389/fcvm.2022.945672. 35990981 PMC9386075

[59] Ayme-Dietrich E , Aubertin-Kirch G, Maroteaux L, Monassier L. Cardiovascular remodeling and the peripheral serotonergic system. Arch Cardiovasc Dis. 2017;110(1):51-59. 10.1016/j.acvd.2016.08.002. 28017279

[60] Laskaratos FM , Davar J, Toumpanakis C. Carcinoid heart disease: a review. Curr Oncol Rep. 2021;23(4):48. 10.1007/s11912-021-01031-z. 33725214

